# *D. russelii* Venom Mediates Vasodilatation of Resistance Like Arteries via Activation of K_v_ and K_Ca_ Channels

**DOI:** 10.3390/toxins11040197

**Published:** 2019-04-01

**Authors:** Rahini Kakumanu, Sanjaya Kuruppu, Lachlan D. Rash, Geoffrey K. Isbister, Wayne C. Hodgson, Barbara K. Kemp-Harper

**Affiliations:** 1 Department of Pharmacology, Biomedicine Discovery Institute, Faculty of Medicine, Nursing & Health Sciences, Monash University, Clayton VIC 3800, Australia; rahini.r.ragavan@monash.edu (R.K.); barbara.kemp@monash.edu (B.K.K.-H.); 2 Department of Biochemistry & Molecular Biology, Biomedicine Discovery Institute, Faculty of Medicine, Nursing and Health Sciences, Monash University, Clayton VIC 3800, Australia; sanjaya.kuruppu@monash.edu; 3 Faculty of Medicine, School of Biomedical Sciences, The University of Queensland, St Lucia QLD 4072, Australia; l.rash@uq.edu.au; 4 Clinical Toxicology Research Group, University of Newcastle, Callaghan NSW 2308, Australia; geoff.isbister@gmail.com

**Keywords:** *D. russelii* venom, hypotension, potassium channels, vasodilatation

## Abstract

Russell’s viper (*Daboia russelii*) venom causes a range of clinical effects in humans. Hypotension is an uncommon but severe complication of Russell’s viper envenoming. The mechanism(s) responsible for this effect are unclear. In this study, we examined the cardiovascular effects of Sri Lankan *D. russelii* venom in anaesthetised rats and in isolated mesenteric arteries. *D. russelii* venom (100 μg/kg, i.v.) caused a 45 ± 8% decrease in blood pressure within 10 min of administration in anaesthetised (100 μg/kg ketamine/xylazine 10:1 ratio, i.p.) rats. Venom (1 ng/mL–1 μg/mL) caused concentration-dependent relaxation (EC_50_ = 145.4 ± 63.6 ng/mL, R_max_ = 92 ± 2%) in U46619 pre-contracted rat small mesenteric arteries mounted in a myograph. Vasorelaxant potency of venom was unchanged in the presence of the nitric oxide synthase inhibitor, L-NAME (100 µM), or removal of the endothelium. In the presence of high K^+^ (30 mM), the vasorelaxant response to venom was abolished. Similarly, blocking voltage-dependent (K_v_: 4-aminopryidine; 1000 µM) and Ca^2+^-activated (K_Ca_: tetraethylammonium (TEA; 1000 µM); SK_Ca_: apamin (0.1 µM); IK_Ca_: TRAM-34 (1 µM); BK_Ca_; iberiotoxin (0.1 µM)) K^+^ channels markedly attenuated venom-induced relaxation. Responses were unchanged in the presence of the ATP-sensitive K^+^ channel blocker glibenclamide (10 µM), or H1 receptor antagonist, mepyramine (0.1 µM). Venom-induced vasorelaxtion was also markedly decreased in the presence of the transient receptor potential cation channel subfamily V member 4 (TRPV4) antagonist, RN-1734 (10 µM). In conclusion, *D. russelii*-venom-induced hypotension in rodents may be due to activation of K_v_ and K_Ca_ channels, leading to vasorelaxation predominantly via an endothelium-independent mechanism. Further investigation is required to identify the toxin(s) responsible for this effect.

## 1. Introduction

Snake bite is a globally important health issue [1,2]. Snake venom has three purposes: a defensive mechanism against predators, an aid to capture prey, and/or a tool to deter/challenge competitors [3]. Venom is a complex cocktail of toxins and enzymes that have a wide range of biological activities targeting major physiological pathways and organs [4]. Ninety to ninety-five percent of snake venom consists of proteins and peptides, many of which are toxic to humans [5]. These components often possess enzymatic activity and ligand binding abilities that, in combination and/or separately, result in the clinical envenoming symptoms in humans and other organisms [3,6]. While most components of snake venom, such as neurotoxins [7,8,9], myotoxins [10,11,12], pro-coagulants, and anticoagulant, haemolytic and local tissue necrotic factors [13,14,15] have been studied in detail, toxins targeting the cardiovascular system are less well understood.

We have previously demonstrated two distinct cardiovascular activities due to snake envenoming: “cardiovascular collapse” (defined as an irreversible rapid drop in blood pressure) [16,17] versus “prolonged hypotension” (defined as a gradual decline in blood pressure that is reversible) [18]. A number of hypotheses have been postulated in regards to the mechanism(s) of “cardiovascular collapse.” This includes the potential involvement of prothrombin activators or pro-coagulant toxins present in snake venoms [19,20]. However, we have previously demonstrated, using an in vivo animal model, that death adder (*A. rugosus*) venom causes collapse even though it contains no pro-coagulant toxins [16]. The release of depletable endogenous mediators has also been postulated to induce “cardiovascular collapse” [16].

Hypotension is defined as a blood pressure that is below the expected normal range for an individual in a given environment. Factors that can affect blood pressure include age, weight, medications, dehydration, or underlying medical conditions. Physiologically, hypotension can occur due to reduced systemic vascular resistance, reduced cardiac output, hypovolemia, vascular obstruction, or blood volume redistribution [21]. Known snake toxins that can affect blood pressure include natriuretic peptides, bradykinin-potentiating peptides, incretin mimetics, and sarafotoxins [22].

In this study, we examined in more detail the hypotensive effects of snake venom and pharmacologically characterized the vasodilatory effects of Sri Lankan Russell’s viper (*D. russelii*) venom. *D. russelii* is regarded as one of the most medically important venomous snakes, as it causes the highest rate of mortality and morbidity due to snake bite in Asia [23,24]. It is mainly found in South Asia and is responsible for 73% of snake envenoming cases in the Anuradhapura District, Sri Lanka [25,26]. Clinical manifestations of envenoming include neuromuscular paralysis, coagulopathy, acute kidney failure, and hypotension [27]. Since the venom consists mainly of neurotoxins and myotoxins (~80%), these toxins have been well characterized pharmacologically [11,24,28], but the cardiovascular effects of this venom are less clear.

## 2. Results and Discussion

*D. russelii* is one of the most medically important species of snakes found in South East Asia, and is responsible for the highest rate of mortality and morbidity in this region due to snake envenoming [28,29,30,31]. The clinical syndromes include coagulopathy, mild neurotoxicity, acute kidney injury, bleeding, and hypotension [11,29,32]. While the neurotoxicity [24,28], acute kidney injury, and coagulopathy [33,34,35] have been studied in great depth, little is known about the cardiovascular effects. In the current study, we investigated the prolonged hypotensive effect of *D. russelii* venom both in vivo and in vitro in rodents.

### 2.1. In Vivo Studies

**Figure 1:**
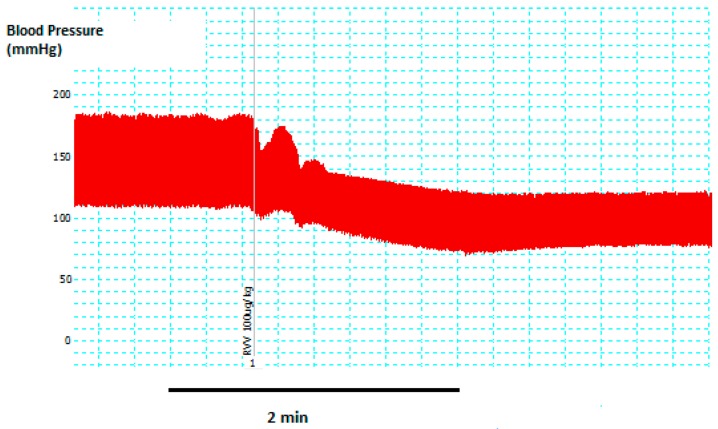
Original recording showing the hypotensive response to *D. russelii* venom (100 g/kg, i.v.) in an anaesthetized rat. Venom (RVV; Russell’s viper venom) was added as indicated by the line on the trace.

*D. russelii* venom (100 µg/kg, i.v., *n* = 4) caused prolonged (30 min) hypotension (45 ± 8% decrease in mean arterial pressure) within 10 min of administration (Figure 1). In addition, the heart rate of rats decreased ~20% i.e. from 289 ± 85 b.p.m. (*n* = 4), just prior to venom administration, to 242 ± 71 b.p.m. (*n* = 4) at the time of maximum decrease in blood pressure.

### 2.2. Vasorelaxant Responses to D. russelii Venom

**Figure 2:**
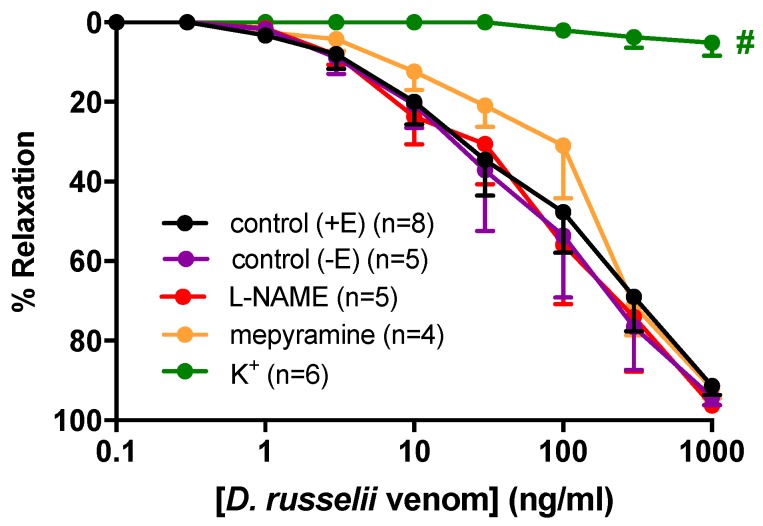
Cumulative concentration-response curves to *D. russelii* venom in rat small mesenteric arteries in the absence or presence of L-NAME (100 µM), 30 mM K^+^, mepyramine (0.1 µM), or following endothelial denudation (−E). Values are expressed as % reversal of U46619 pre-contraction and given as mean ± SEM, where *n* = number of animals. ^#^ *p* < 0.05, response at 1000 ng/mL versus control (+E) (one-way ANOVA, Bonferonni’s post-hoc test).

We next identified *D. russelii* venom as a potent dilator (EC_50_ = 145.4 ± 63.6 ng/mL, R_max_ = 92 ± 2%; Figure 2) of isolated rat small mesenteric arteries. Interestingly, vasorelaxation was unchanged following endothelial denudation (EC_50_ = 137.6 ± 51.6 ng/mL, R_max_ = 87 ± 7%) or inhibition of nitric oxide synthase by L-NAME (100 µM, EC_50_ = 130.7 ± 72.5 ng/mL, R_max_ = 91 ± 5%), indicating that the venom is likely directly targeting the vascular smooth muscle to cause relaxation. Thus, we next sought to characterize the mechanism(s) via which the venom mediates endothelium-independent vasorelaxation.

In the presence of the histamine H1 receptor antagonist, mepyramine (0.1 µM), there was no change in vasorelaxation (EC_50_ = 159.4 ± 82.3, R_max_ = 76 ± 10%) compared to venom alone, indicating that histamine does not appear to play a role in *D. russelii*-venom-induced vasorelaxation. In contrast, raising the extracellular concentration of K^+^ to 30 mM abolished the vasorelaxation effects of venom (R_max_ = 5 ± 3%, *p* < 0.05, Table 1), suggesting that the venom also modulates relaxation of small resistance-like arteries in part via activation of K^+^ channels.

### 2.3. Contribution of Potassium Channels to D. russelii-Mediated Vasorelaxation

It is well known that potassium channels play an integral role in maintaining the membrane potential and therefore, contractile tone in smooth muscle cells [36]. The distribution and nature of potassium channels vary depending upon the size of the vessel, as well as the type of vascular bed [37,38]. There are at least four different subtypes of potassium channels present in mesenteric arterial smooth muscle cells. These include inward rectifier (K_IR_), voltage-gated (K_V_), ATP sensitive (K_ATP_), and Ca^2+^-activated (K_Ca_) potassium channels [36,39]. When K^+^ channels are activated, it leads to vascular smooth muscle cell hyperpolarization and relaxation, thereby leading to a decrease in blood pressure and increased blood flow [36].

Whilst there are many different venoms that are known to contain potassium channel inhibiting peptides, the current study has identified an apparent ability of *D. russelii* venom to activate potassium channels.

**Figure 3:**
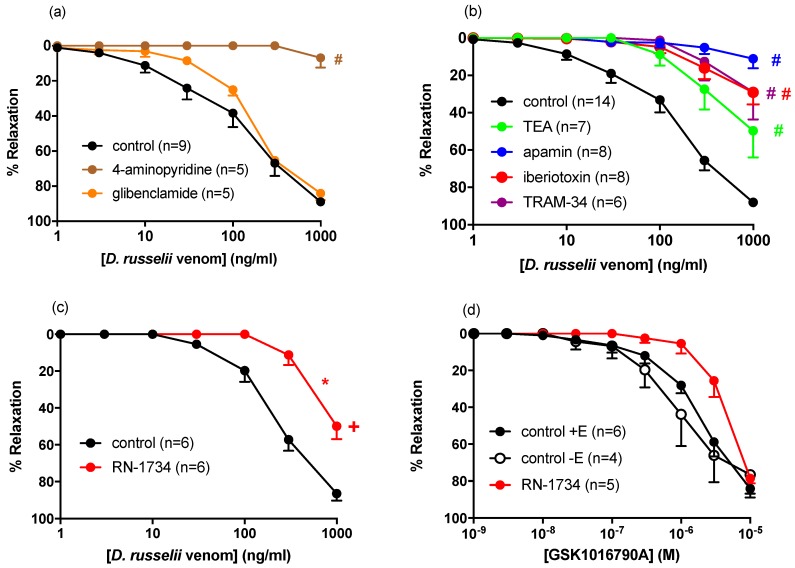
Cumulative concentration–response curves to *D. russelii* venom in rat small mesenteric arteries in the absence or presence of (**a**) 4-aminopyridine (1000 µM) or glibenclamide (10 µM), (**b**) TEA (1000 µM), apamin (0.1 µM), iberiotoxin (0.1 µM) or TRAM-34 (1 µM), or (**c**) RN-1734 (10 µM). (**d**) Cumulative concentration–response curves to GSK1016790A in the absence (control +E) or presence of RN-1734 (10 µM) or in endothelium-denuded vessels (control −E). Values are expressed as % reversal of pre-contraction and given as mean ± SEM, where *n* = number of animals. # *p* < 0.05, response at 1000 ng/mL versus control (one-way ANOVA, Bonferonni’s post-hoc test), + *p* < 0.05, response at 1000 ng/mL versus control (student’s unpaired t-test), * *p* < 0.05 vs control concentration-response curve (two-way repeated measures ANOVA).

Vasorelaxation to *D. russelii* venom in rat small mesenteric arteries was unchanged in the presence of the ATP-sensitive K^+^ channel inhibitor, glibenclamide (10 µM; EC_50_ = 237.7 ± 62.1, R_max_ = 84 ± 4%, Figure 3a). However, the voltage-gated K^+^ channel inhibitor 4-aminopyridine, (1000 µM) abolished venom-induced relaxation, reducing the response at 1000 ng/mL to 7 ± 6% (*p* < 0.01; Table 1). The non-selective K_Ca_ channel blocker TEA, (1000 µM, R_max_ = 50 ± 14%), markedly attenuated venom-induced vasorelaxation. Similarly, blocking large (BK_Ca_; iberiotoxin, 0.1 µM, R_max_ = 29 ± 6%), intermediate (IK_Ca_; TRAM-34 1 µM, R_max_ = 29 ± 15%), and small (SK_Ca_; apamin, 0.1 µM, R_max_ = 11 ± 5%, Figure 3b, Table 1) Ca^2+^-activated K^+^ channels significantly inhibited venom-induced vasorelaxation.

These findings suggest the involvement of both K_Ca_ and K_v_ channels in *D. russelii*-venom-induced vasorelaxation. Given both K_Ca_ and K_v_ channels are sensitive to Ca^2+^, our observations raise the interesting possibility that the venom may modulate intracellular Ca^2+^ levels, thereby indirectly modulating K^+^ channel function. Indeed, our observation that the vasorelaxant effect of the venom was attenuated in the presence of the TRPV4 antagonist, RN-1734 (10 µM), further supports this concept. Specifically, in the presence of RN-1734, the potency of the venom was decreased approximately five-fold, and the response to 1000 ng/mL significantly reduced from 86 ± 4 to 50 ± 7% (Figure 3c). The TRPV4 agonist, GSK1016790A, also caused concentration-dependent relaxation (EC_50_ = 2.8 ± 0.7 µM, R_max_ = 84 ± 5%) in rat small mesenteric arteries, a response which was unchanged following endothelial denudation (EC_50_ = 1.3 ± 0.6 µM, R_max_ = 77 ± 10%). In the presence of RN-1734 (10 µM), there was an apparent two-fold decrease in the potency of GSK1016790A, yet this change failed to reach statistical significance (EC_50_ = 3.5 ± 0.9 µM, Figure 3d, Table 1). The greater inhibitory effect of RN-1734 against *D. russelii*-venom-induced vasorelaxation, as compared to GSK1016790A, may reflect a lower efficacy of the venom as a TRPV4 activator or an ability of RN-1734 to target TRPV4-independent signaling pathways potentially activated by the venom, identification of which are beyond the scope of the current study.

Whilst TRPV4 receptors are abundantly expressed on the endothelium [40], they are also present on the vascular smooth muscle [40,41]. Here, activation of TRPV4 leads to Ca^2+^ influx, the generation of Ca^2+^ sparks from the sarcoplasmic reticulum, and the subsequent activation of BK_Ca_ and vasorelaxation [42,43]. As such, *D. russelii* venom may cause vasorelaxation of resistance arteries in part via activation of vascular smooth muscle TRPV4 and opening of K_Ca_. Future patch-clamp studies are required to support this hypothesis, and the potential for the venom to directly activate K_Ca_ and K_v_ channels remains.

## 3. Conclusions

*D. russelii* venom is known to cause an array of clinical manifestations in envenomed patients. These include coagulopathy, mild neurotoxicity, acute kidney injury, and sometimes severe hypotension. In this study, we have demonstrated that the hypotensive response may be indicative of a vasodilatory action of the venom at the level of the resistance vasculature. We have shown that *D. russelii* venom is a potent dilator of resistance-like arteries, mediating its response via activation of K_v_ and K_Ca_ channels, which may be modulated, in part, via signaling downstream of TRPV4. Future studies, including electrophysiological experiments and separation of venom components using HPLC, as well as investigating the potential contribution of other potassium channels such as K_IR_ channels and Na^+^/K^+^-ATPase, will aid in identifying the toxin(s) responsible for relaxation and provide further insight into the underlying mechanisms.

## 4. Materials and Methods

### 4.1. In Vivo Blood Pressure Experiments

Animal experiments were approved by the Monash University Ethics Committee (MARP/2014/097). Sprague-Dawley male rats (weight 280–350g) were anaesthetized with a mixture of ketamine (100 mg/kg, i.p.) and xylazine (10 mg/kg, i.p.). A midline incision was made and cannulae inserted into the trachea for mechanical ventilation, if required. Cannulae were also inserted into the left jugular vein for administration of venom and the right carotid artery to record arterial blood pressure. The arterial cannula was connected to a pressure transducer (PowerLab/400 system, ADInstruments Inc, Sydney, NSW, Australia). Blood pressure was then allowed to stabilize for approximately 10–15 min. Body temperature was maintained at 37 °C using an overhead lamp and heated rat table. Venom (100 µg/kg) was administrated via the jugular vein followed by flushing with saline. Responses to the venom were measured as percentage change in mean arterial pressure (MAP).

### 4.2. Isolation of Rat Small Mesenteric Arteries

Male Sprague-Dawley rats (200–250g) were euthanized via CO_2_ inhalation (95% CO_2_, 5% O_2_), followed by exsanguination. Small mesenteric arteries (second-order branch of the superior mesenteric artery) were isolated, cut into 2 mm lengths, and mounted on 40 µm wires in small vessel myographs [44]. Vessels were maintained in physiological salt solution (composed of (in mM) 119 NaCl, 4.7 KCl, 1.17 MgSO_4_, 25 NaHCO_3_, 1.8 KH_2_PO_4_, 2.5 CaCl_2_, 11 glucose and 0.026 EDTA) at 37 °C, and were bubbled with carbogen (95% O_2_, 5% CO_2_). In a subset of arteries, the endothelium was gently denuded via insertion of a 40 µm wire inside the lumen and rubbing the vessel walls. The mesenteric arteries were allowed to equilibrate for 30 min under zero force and then a 5 mN resting tension was applied. Changes in isometric tension were recorded using Myograph Interface Model 610 M v2.2 (DMT, Aarhus, Denmark) and PowerLab/835 (ADInstruments Inc). Data were recorded with the data acquisition program Chart (v5, ADInstruments). Following a 30 min equilibration period at 5 mN, the mesenteric arteries were contracted maximally (F_max_) using a K^+^ depolarizing solution (K^+^-containing physiological salt solution (KPSS); composed of (in mM) 123 KCl, 1.17 MgSO_4_, 1.18 KH_2_PO_4_, 2.5 CaCl_2_, 25 NaHCO_3_ and 11 glucose). The integrity of the endothelium was confirmed by relaxation to acetylcholine (ACh, 10 µM) in tissues pre-contracted with the thromboxane A_2_ mimetic, U46619 (1 µM). Vessels with a relaxation response to ACh < 20% were considered endothelium-denuded, while vessels with a relaxation response to ACh > 80% were considered endothelium-intact. Arteries were washed with physiological salt solution and the tension was allowed to return to baseline.

### 4.3. Vasorelaxation Experiments

Cumulative concentration–response curves to venom (1 ng/mL–1 µg/mL) were constructed in vessels pre-contracted submaximally to ~50% F_max_ with titrated concentrations of U46619 (0.01–0.2 µM). Responses to venom were obtained in endothelium-intact mesenteric arteries pre-incubated for 30 min with either 30 mM K^+^ [45], mepyramine (0.1 µM), L-NAME (100 µM) [44], TEA (1000 µM) [46], iberiotoxin (0.1 µM), apamin (0.1 µM), TRAM-34 (1 µM), 4-aminopyridine (1000 µM) [44], glibenclamide (10 µM) [47,48] or RN-1734 (10 µM). Cumulative concentration-response curves were also constructed to venom alone (1 ng/mL–1 µg/mL) or GSK1016790A in the presence and absence of RN-1734 (10 µM). In a subset of endothelium-denuded arteries, the vasorelaxation of venom and GSK1016790A were also examined. Sodium nitroprusside (SNP; 10 µM) [49] was added at the end of each concentration response curve to ensure maximum relaxation. Only one concentration–response curve to venom was obtained in each vessel segment due to tachyphylaxis [49,50].

### 4.4. Data Analysis and Statistical Procedures

#### 4.4.1. In Vivo

For the anaesthetized rat experiments, pulse pressure was defined as the difference between systolic and diastolic blood pressures. Mean arterial pressure (MAP) was calculated as diastolic blood pressure plus one-third of pulse pressure. Heart rate (HR) was determined from the blood pressure trace.

#### 4.4.2. In Vitro

Blood vessel relaxation was expressed as a percentage reversal of the U46619 pre-contraction (i.e., cumulative relaxation responses to venom or GSK1016790A were measured as a change in tension from the stable U46619 contraction, and expressed as a percentage of this contractile response). Individual relaxation curves were fitted to a sigmoidal logistic equation and EC_50_ values (concentration of agonist resulting in a 50% relaxation) were calculated. Statistical comparisons between the experimental groups’ mean EC_50_ and maximum relaxation (R_max_) values were made using either a student’s unpaired t-test or a one-way ANOVA with Bonferroni’s post hoc comparison. Where EC_50_ values could not be obtained, concentration–response curves were compared by means of a two-way ANOVA (repeated measures). *n* = Number of artery segments from separate animals. Data represent the mean ± SEM (error bars on graph). Statistical significance was defined as *p* < 0.05. All data analysis was performed using GraphPad Prism v5.02 (GraphPad Software, San Diego, CA, USA) [44].

### 4.5. Reagents

Reagents and their sources were: U46619 (Cayman Chemical company, Ann Arbor, MI, USA), glibenclamide, TEA, 4-aminopyridine, TRAM-34, mepyramine, L-NAME, SNP, isoprenaline, ACh, RN-1734 and GSK1016790A (Sigma-Aldrich, St Louis, MO, USA), and iberiotoxin (In vitro Technologies, Melbourne, VIC, Australia). Stock solutions of U46619 (1 mM) were made up in absolute ethanol. All subsequent dilutions of stock solutions were in distilled water. All other drugs were dissolved in distilled water, and all dilutions were prepared fresh daily.

## Figures and Tables

**Table 1:** Effect of treatments on *D. russelii*- or GSK1016790A-induced vasorelaxation in rat small mesenteric arteries.

Treatment	*D. russelii* Venom		
	EC_50_ (ng/mL)	R_max_ (%)	*n*
**Control (+E)**	145.4 ± 63.6	92 ± 2	8
**Control (−E)**	137.6 ± 51.6	87 ± 7	5
**L-NAME**	130.7 ± 72.5	91 ± 5	5
**Mepyramine**	159.4 ± 82.3	76 ± 10	4
**K^+^**	ND	5 ± 3 ^#^	5
**Control**	276.5 ± 69.3	88 ± 2	15
**TEA**	ND	50 ± 14 ^#^	7
**Apamin**	ND	11 ± 5 ^#^	8
**Iberiotoxin**	ND	29 ± 6 ^#^	8
**TRAM-34**	ND	29 ± 15	6
**Control**	328.7 ± 110.5	89 ± 2	9
**Glibenclamide**	237.7 ± 62.1	84 ± 4	4
**4-Aminopyridine**	ND	7 ± 6 ^#^	5
**Control**	273.6 ± 57.6	86 ± 4	6
**RN-1734**	ND	50 ± 7 *	6
	**GSK1016790A**		
**Control (+E)**	2.8 ± 0.7 µM	84 ± 5	6
**Control (−E)**	1.3 ± 0.6 µM	77 ± 10	4
**RN-1734**	3.5 ± 0.9 µM	79 ± 3	5

Values as % reversal of the level of pre-contraction; +E = endothelium intact; −E = endothelium denuded; ND = Not determined; ^#^ *p* < 0.05, 1-way ANOVA as compared to control * *p* < 0.05, student’s unpaired t-test.
